# Harnessing phages to tackle antimicrobial resistance: a Saudi Arabian perspective

**DOI:** 10.3389/fcimb.2025.1702890

**Published:** 2025-11-20

**Authors:** Alhassan Alrafaie

**Affiliations:** Department of Medical Laboratory, College of Applied Medical Sciences in Al-Kharj, Prince Sattam Bin Abdulaziz University, Al-Kharj, Saudi Arabia

**Keywords:** bacteriophage, antimicrobial resistance, phage therapy, Saudi Arabia, antibiotic

## Abstract

The worldwide antimicrobial resistance (AMR) threat continues to grow, causing an estimate of 4.71 million deaths during 2021. Scientists predict it could lead to 10 million deaths each year by 2050 unless effective solutions are developed. The government of Saudi Arabia implemented the AMR Action Plan (2022–2025) and participated in the 4th Global High-Level Ministerial Conference on AMR (2024) to address this issue. However, ESBL-producing Enterobacteriaceae and carbapenem-resistant *Acinetobacter baumannii* remain major threats in Saudi Arabia. Bacteriophage therapy offers a promising additional treatment approach as phages specifically attack bacteria. They also evolve and demonstrate effectiveness against bacteria that form biofilms. This review evaluates Saudi Arabia’s readiness for phage-based therapy implementation through an analysis of both local and worldwide antibiotic resistance data.

## 1 Introduction

The 21st century faces antimicrobial resistance (AMR) as its most significant worldwide health danger which attacks contemporary medical practices and public health organizations. The fast development of antibiotic-resistant bacteria has made traditional antibiotics less effective which creates major challenges for treating infections and leads to higher rates of illness and death. Research studies in Saudi Arabia show that antibiotic resistance levels are high in Gram-positive and Gram-negative bacteria which demands immediate intervention to control transmission and create new therapeutic solutions (Alqasim et al., 2018; Somily et al., 2021; Alshammari et al., 2023). Scientists now focus on antimicrobial peptides, probiotics, fecal microbiota transplants and bacteriophage-based methods as new antibacterial treatments since existing antibiotics are losing their effectiveness (Singha et al., 2024). Bacteriophages which are viruses that target bacteria exist everywhere in nature and show promise as alternative therapy as they target bacteria precisely while multiplying on their own (Molineux and Panja, 2013). This review presents an overview of worldwide and Saudi Arabian AMR patterns while explaining bacteriophage therapy principles and assessing its benefits and obstacles for Saudi healthcare organizations.

## 2 Antimicrobial resistance crisis

**Figure 1:**
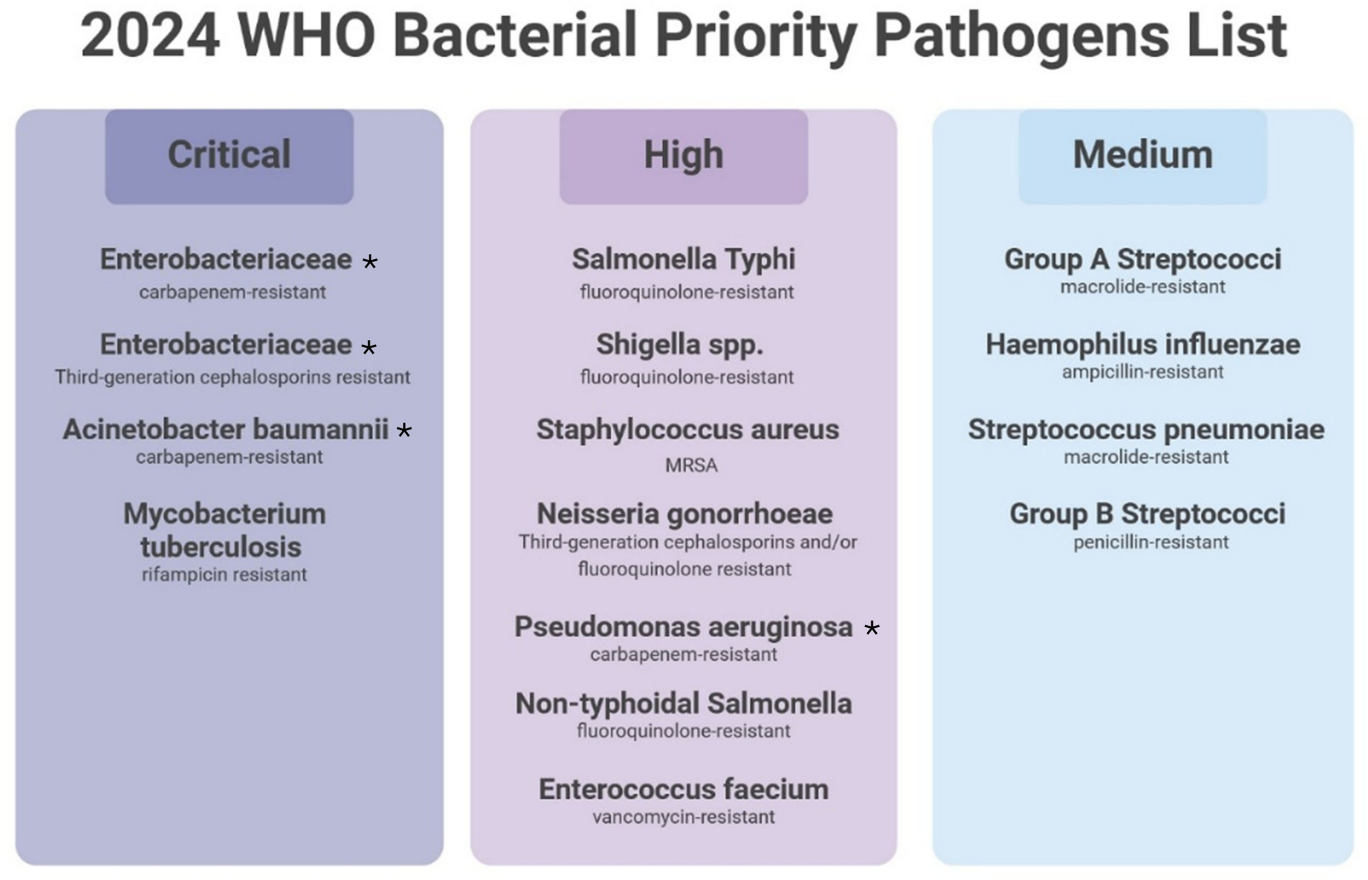
2024 WHO bacterial priority pathogens list. Pathogens are grouped into Critical, high or medium priority levels. Pathogens nomenclatures with antibiotics resistance are shown. MRSA: Methicillin-resistant *Staphylococcus aureus*. Asterisks refer to pathogens prevalent in Saudi Arabia. Created in BioRender http://www.biorender.com.

Antimicrobial resistance arises from both improper medication practices and excessive antibiotic use in medical treatments and agricultural practices (Kushwaha et al., 2024). The use of antibiotics in animal husbandry, along with improper prescriptions, has also contributed to the development and spread of resistant bacterial strains (Talebi Bezmin Abadi et al., 2019). Consequently, the emergence of antibiotic resistance prolongs illness durations, as well as increasing death rates and healthcare expenditures (Dadgostar, 2019). In 2021 alone, AMR was associated with 4.71 million deaths worldwide (Naghavi et al., 2024). Recently, The WHO introduced an advanced Global Antimicrobial Resistance and Use Surveillance System (GLASS) dashboard during September 2025 which showed concerning data from 141 participating nations (WHO, 2025a). The system tracks more than 23 million bacteriologically confirmed infection cases from 110 countries during 2016–2023 and tracks resistance development in eight major bacterial pathogens and 23 antibiotics. Moreover, the Global Antibiotic Resistance Surveillance Report 2025 expands on these results by presenting complete worldwide and regional assessments of resistance patterns showing one in six bacterial infections globally is resistant to standard antibiotics (Global antibiotic resistance surveillance report 2025, n.d). Beyond its health impact, this public health concern presents catastrophic economic consequences, with experts forecasting losses that could reach $100 trillion globally by 2050 if the current crisis remains unaddressed (O’neill, 2016). The financial burden grows through higher medical costs and longer durations of hospitalization (Kushwaha et al., 2024). Moreover, the emergence of multidrug-resistant bacteria presents an urgent global health challenge, leading to a “post-antibiotic era” in which numerous infections may become nearly impossible to treat (Hauser et al., 2016) (**Table 1**). Therefore, the current crisis requires urgent collaborative actions to maintain the effectiveness of existing antibiotics while researching new medical solutions (Aslam et al., 2018). However, the slow progress in antibiotic development has intensified the crisis, leaving new treatment options scarce. To overcome this challenge, research must now focus on developing non-antibiotic treatment strategies as alternatives for treating resistant bacterial infections (Hauser et al., 2016). In response to these growing threats, the world health organization (WHO) updated its Bacterial Priority Pathogens List in 2024 to address the evolving AMR threats (WHO, 2024). The new list categorizes 15 pathogens into critical, high, and medium priority tiers based on 4 main criteria: clinical impact (mortality/morbidity), resistance burden, transmissibility, and treatment complexity (**Figure 1**).

**Table 1:** Summary of antibacterial classes, cellular targets, and key resistance mechanisms.

Drug class (examples)	Primary target(s)/ accession no	Cellular localization	Mode of action (MOA)	Common resistance mechanisms / mutations (examples)	References
β-lactams (penicillins, cephalosporins, carbapenems)	PBP1A (P02918); PBP2 (P0AD65);	Inner membrane	Inhibit transpeptidation which blocks peptidoglycan cross-linking	Target alteration (PBP2a via mecA in MRSA); β-lactamases (ESBLs CTX-M-15, KPC, NDM, OXA-48); porin loss (OmpK35/36); increased efflux	(Bush and Bradford, 2016)
Glycopeptides (vancomycin)	Enolpyruvate transferase MurA (P0A749); D-Ala–D-Ala ligase DdlB (P07862) VanA ligase (P25051)	Cytoplasmic (MurA/DdlB); membrane-associated (VanA)	Bind to D-Ala-D-Ala and prevent transglycosylation and transpeptidation	Van operons (vanA/vanB) cause D-Ala-D-Lac replacement reducing binding	(Mareković et al., 2024)
Lipopeptides (daptomycin)	Lysyl-phosphatidylglycerol synthase/flippase MprF (Q2G2M2) Cardiolipin synthase A ClsA (P0A6H8)	Cytoplasmic membrane	Use calcium-dependent membrane insertion leading to membrane depolarization	mprF mutations; yycG alterations; increased cell surface charge repels the drug	(Taylor and Moreira, 2025)
Polymyxins (colistin)	Lipid A biosynthesis deacetylase LpxC (P0A725)	Cytoplasmic membrane	Disrupt the outer membrane through binding to lipopolysaccharide	LPS modification (pmrA/pmrB; mgrB inactivation); plasmid-mediated mcr-1 to mcr-10	(Mondal et al., 2024)
Fluoroquinolones (ciprofloxacin, levofloxacin)	DNA gyrase subunit A GyrA (P0AES4); Topoisomerase IV subunit A ParC (P0AFI2)	Cytoplasm	Stabilize the DNA–enzyme cleavage complex and cause double-stranded DNA breaks	gyrA S83L/D87N; parC S80I; efflux (AcrAB-TolC); qnr genes	(Kherroubi et al., 2024)
Aminoglycosides (amikacin, gentamicin)	30S ribosomal protein S12 / RpsL (P0A7S3)	Cytoplasm	Induce misreading and inhibit protein translation	Aminoglycoside-modifying enzymes (aac(6')-Ib, aph, ant); 16S rRNA methylases (armA, rmtB)	(Wachino et al., 2020)
Tetracyclines (doxycycline, tigecycline)	30S ribosomal protein S10 / RpsJ (P0A7R5)	Cytoplasm	Block aminoacyl-tRNA binding to the ribosome	Efflux pumps (tetK/A); ribosomal protection proteins (tetM/O); enzymatic inactivation (tetX)	(Pearson et al., 2025)
Macrolides (azithromycin, erythromycin)	50S ribosomal proteins L4 / RplD (Q2FW07), L22 / RplV (Q2FW11)	Cytoplasm	Block the peptide exit tunnel and inhibit elongation	23S rRNA A2058G mutation; methylation by erm genes; mef-mediated efflux	(Nor Amdan et al., 2024)
Oxazolidinones (linezolid)	50S ribosomal protein L3 / RplC (Q2FW06)	Cytoplasm	Inhibit initiation and peptidyl transferase activity	23S rRNA G2576T; cfr methyltransferase (A2503)	(Peykov et al., 2025)
Rifamycins (rifampicin)	DNA-directed RNA polymerase β subunit / RpoB (P0A8V2)	Cytoplasm	Block transcription initiation	rpoB S531L and H526Y mutations	(Bolourchi et al., 2025)
Folate antagonists (trimethoprim–sulfamethoxazole)	DNA-directed RNA polymerase β subunit / RpoB (P0A8V2)	Cytoplasm	Block folate synthesis at two sequential steps	dfrA variants; sul1 and sul2 genes	(Wróbel et al., 2019)

## 3 Strategies to overcome multidrug resistance

The fight against multidrug-resistant (MDR) pathogens demands coordinated strategies to combat infections and prevent the emergence of new drug-resistant strains. The two primary strategies to preserve antibiotic effectiveness consist of antimicrobial-stewardship programs and infection-prevention and control (IPC) measures (Core Elements of Antibiotic Stewardship | Antibiotic Prescribing and Use | CDC; Global report on infection prevention and control 2024). These strategies aim to enhance antibiotic use while building up surveillance capabilities and evidence-based prescribing methods to reduce antibiotic misuse. The One Health approach receives support from worldwide and national programs which integrate human, animal, environmental health efforts to tackle antibiotic resistance and preserve antibiotic effectiveness (Musicha et al., 2024).

The first line of antibacterial treatments serve as the base for initial treatment approaches and function as a critical factor in controlling antibiotic resistance development. These treatments include β-lactams (amoxicillin–clavulanate and ceftriaxone), fluoroquinolones (ciprofloxacin and levofloxacin), macrolides (azithromycin and clarithromycin) and aminoglycosides (gentamicin and amikacin) (Moja et al., 2024). These antibiotics are categorized within the WHO AWaRe framework as essential options for empiric management of common community and hospital infections. The goal of their use is to provide equal access to treatment while reducing drug resistance through evidence-based prescribing, proper treatment duration and local adaptation of treatment guidelines.

Preventive interventions function as critical measures which help reduce bacterial infections while controlling the spread of multidrug-resistant (MDR) pathogens. The combination of improved infection control methods, expanded vaccination programs and proper prophylactic treatment delivery leads to reduced antibiotic use and lower bacterial resistance to antibiotics (WHO, 2025b). Additionally, WHO supports intermittent preventive therapy (IPT) as an effective preventive chemotherapeutic method which shows that planned drug delivery reduces infection rates (WHO, 2022). The implementation of IPT-like methods for bacterial infections together with vaccination and IPC practices will enhance antimicrobial stewardship programs while maintaining control of MDR pathogens through a One Health framework (Sartelli et al., 2024).

Biological therapies function as additional treatments that work alongside traditional antibiotic medications. Bacteriophage therapy provides distinct strategies to target bacteria through phage-mediated cell burst and through the combination of phages with antibiotics. Moreover, phage-derived endolysins and other bacteriolytic enzymes work together to dissolve biofilms which helps eliminate treatment-resistant pathogens (Liu et al., 2023; Khan et al., 2024). The development of antimicrobial peptides and anti-virulence agents that interfere with quorum sensing and toxin production also offers alternative non-antibiotic therapeutic approaches (Fontanot et al., 2024; Defoirdt, 2025). The management of MDR infections needs a unified healthcare system which integrates drug-based treatments with protective measures and biological treatment approaches.

## 4 Bacteriophages

Frederick Twort first discovered bacteriophages (bacterial viruses or phages) in 1915 followed shortly by Félix d’Hérelle in 1917 (Twort, 1915; Lourenço et al., 2018). The discovery of bacteriophages represented a significant breakthrough in microbiology, leading to their use in treating bacterial infections, which means phage therapy and bacterial identification via phage typing (Letarov, 2020). However, the emergence of antibiotics in the 1940s limited phage therapy and confined its use to countries such as Poland and Georgia (Letarov, 2020).

The structure of bacteriophages, like other viruses, consists mainly of a protein shell surrounding nucleic acid molecules, which can be either DNA or RNA. The morphology of bacteriophages can be determined using transmission electron microscopy, allowing for their classification into tailed or non-tailed virions. Tailed phages belong to the *Caudoviricetes* class and are morphologically classified into 3 categories: myovirus (long contractile tails), siphovirus (long non-contractile tails), and podovirus (short tails) (Broeker et al., 2019). These tailed phages are abundant and comprise the vast majority of identified phages. Non-tailed phages can be pleomorphic, filamentous, polyhedral or icosahedral virions (Ackermann, 2007). It is important to note that these morphological terms refer to phage morphology but not classification, as the current International Committee on Taxonomy of Viruses recommendations emphasize bacteriophage classification based on genetic information (Krupovic et al., 2021).

**Figure 2:**
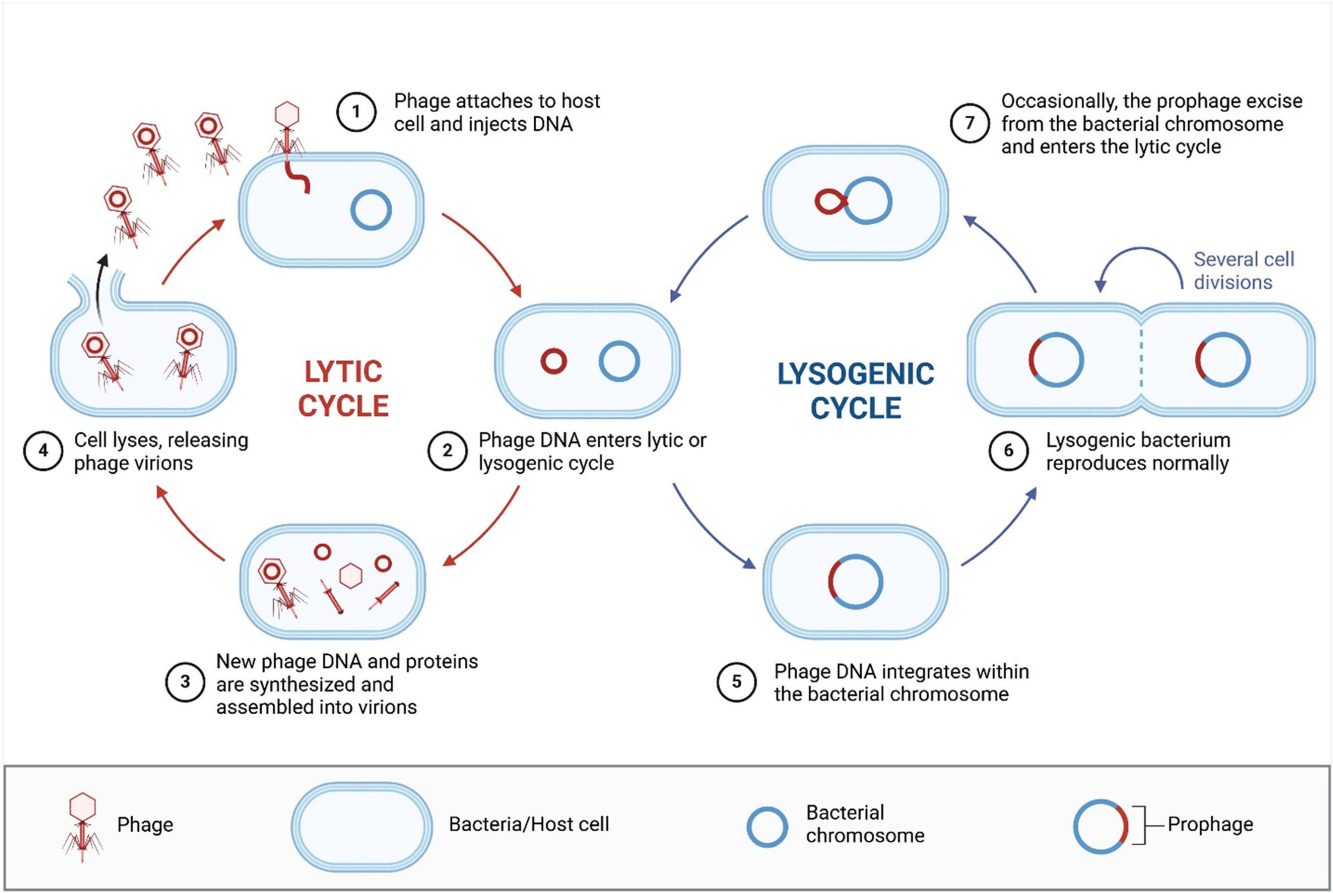
Phage lytic and lysogenic lifecycles. (1) Phages first adsorb onto bacterial cells and DNA is injected into the cell. (2) phage DNA undergoes either lytic or lysogenic. (3) In lytic lifecycle, phages take over the cell and phage DNA and proteins are synthesized and assembled. (4) phages are released via lysing the bacterial cell. (5) For lysogenic lifecycle, Phage DNA in integrated into host chromosome and called prophage. (6) lysogenic bacterium reproduces normally. (7) under certain conditions, prophage genomes can be induced and then enters the lytic cycle. Created in BioRender http://www.biorender.com.

Regarding lifecycles, bacteriophages exhibit 2 main types: lytic and lysogenic. During the lytic cycle, phages first adsorb onto the bacterial cell, targeting specific receptors and then inject their viral genome into the bacterial cell. The phage then takes over the infected cell, leading to the biosynthesis of new phage particles, which are released via cell burst (Sharma et al., 2017). In the lysogenic cycle, phage DNA integrates with the host bacterial genome and remains in a dormant state (prophage) without progeny production. The prophage can be induced, allowing the phage genome to switch to the lytic cycle under specific conditions (**Figure 2**).

## 5 Bacteriophage therapy

Bacteriophage therapy involves exploiting bacteriophages to treat bacterial infections, a strategy that has gained prominence due to the rise of antibiotic-resistant bacteria. The need for alternative treatments has made bacteriophage therapy a viable option for combating antibiotic resistance. To target a specific pathogen, one or more phages can be used in the treatment regimen. In this context, phage cocktails (mixtures of 2 or more phages) are employed to enhance the efficiency of phage therapy by providing wide-spectrum coverage against multiple bacterial strains. Moreover, such cocktails can minimize bacterial resistance compared to single-phage therapy. However, one challenge with the phage cocktail approach is balancing complexity and efficacy, as excessive complexity may result in dysbiosis and increased production costs (Molina et al., 2021). To address this challenge, enriched combinations of phages can increase the efficacy of the cocktails, and including functional diversity may enhance their effectiveness even with fewer combinations (Wright et al., 2021). Furthermore, phage cocktails can be strategically designed to remain effective despite the evolutionary adaptations of bacteria, which confer resistance (Yoo et al., 2024).

In addition to whole phage particles, phage enzymes can be exploited to attack and lyse bacterial hosts. Phage-encoded enzymes have been considered potential weapons against bacteria as antibiotics lose their effectiveness. These enzymes fall into 3 main categories: endolysins, virion-associated lysins (VALs), and polysaccharide depolymerases. Each type has its own structure and mechanism of action.

Endolysins are integral enzymes that phages utilize to release virions from bacterial cells. These endolysins have 2 main parts: an N-terminal enzymatically active domain (EAD) and a C-terminal cell wall binding domain (CBD). The EAD breaks various bonds in the bacterial cell wall (peptidoglycan), acting as an amidase, muramidase, or endopeptidase (Jarábková et al., 2015). The CBD targets specific bacterial cells, ensuring efficiency and reducing off-target effects. During the phage lifecycle, endolysins break down the bacterial cell wall from within to allow the release of new phage particles. Importantly, regarding phage therapy, endolysins can also break down bacterial cell walls when applied externally on target bacteria, making them useful as standalone antibacterial agents (Abdelrahman et al., 2021). In contrast to endolysins, VALs facilitate the phage lifecycle by creating tiny holes in the bacterial cell wall, allowing phage DNA to enter the host cell, thus participating in the initial steps of the phage lifecycle (Rodríguez-Rubio et al., 2013). The VALs EAD can act as an amidase, muramidase, or endopeptidase, similar to endolysins, targeting peptidoglycan. VALs can degrade other targets on the bacterial surface, such as teichoic acid, using Glycerophosphodiester Phosphodiesterases in Gram-positive bacteria (Alrafaie and Stafford, 2022). VALs exhibit excellent specificity and can withstand high temperatures, making them promising candidates for antibacterial applications (Latka et al., 2017). As for depolymerases, they target extracellular polysaccharides, such as bacterial capsules and biofilm matrices, facilitating the degradation of protective barriers. This enables phages to effectively target biofilms and work synergistically with other antimicrobial agents (Topka-Bielecka et al., 2021).

For the treatment of antibiotic-resistant bacteria, phages can be combined with antibiotics to enhance treatment effectiveness or manage resistance more effectively. The effectiveness of phage-antibiotic combination therapy has been evidenced in both *in vitro* and *in vivo* studies (Kaczorowska et al., 2021). Importantly, the success of such combinations depends significantly on the antibiotics used, as certain agents can antagonize phage activity. For instance, antibiotics targeting bacterial protein synthesis pathways might reduce phage lytic activity, indicating the need for careful antibiotic selection in combination therapy (Vashisth et al., 2022). Research also shows that phage-encoded enzymes synergize with other antimicrobials, such as antimicrobial peptides, to increase their therapeutic efficacy against antibiotic-resistant bacterial biofilms (Tyagi et al., 2024). Studies have also demonstrated that recombinant depolymerases act as antibiotic adjuvants, making multidrug-resistant *A. baumannii* more susceptible to immune system attacks and enhancing the bactericidal effects of antibiotics such as colistin (Chen et al., 2022). Moreover, combining phage endolysins with antimicrobial peptides has shown a synergistic effect against bacterial biofilms, highlighting the promise of combination therapy to fight antibiotic-resistant bacterial infections (Tyagi et al., 2024). Overall, this combined regimen can lower bacterial virulence and enhance bacterial susceptibility to antibiotics (Anastassopoulou et al., 2024).

Besides natural phages, advances in genetic engineering have greatly increased the potential for developing engineered phages with improved therapeutic potential. These phages could be designed to actively lyse antibiotic-resistant bacteria or to carry genes promoting the degradation of bacterial biofilms and the production of antimicrobial peptides, further enhancing their efficacy (Hibstu et al., 2022; Olawade et al., 2024). The engineered phages can also enable highly personalized phage therapy, customized specifically for the bacterial infections of individual patients (Dedrick et al., 2019; Hou et al., 2025).

**Figure 3:**
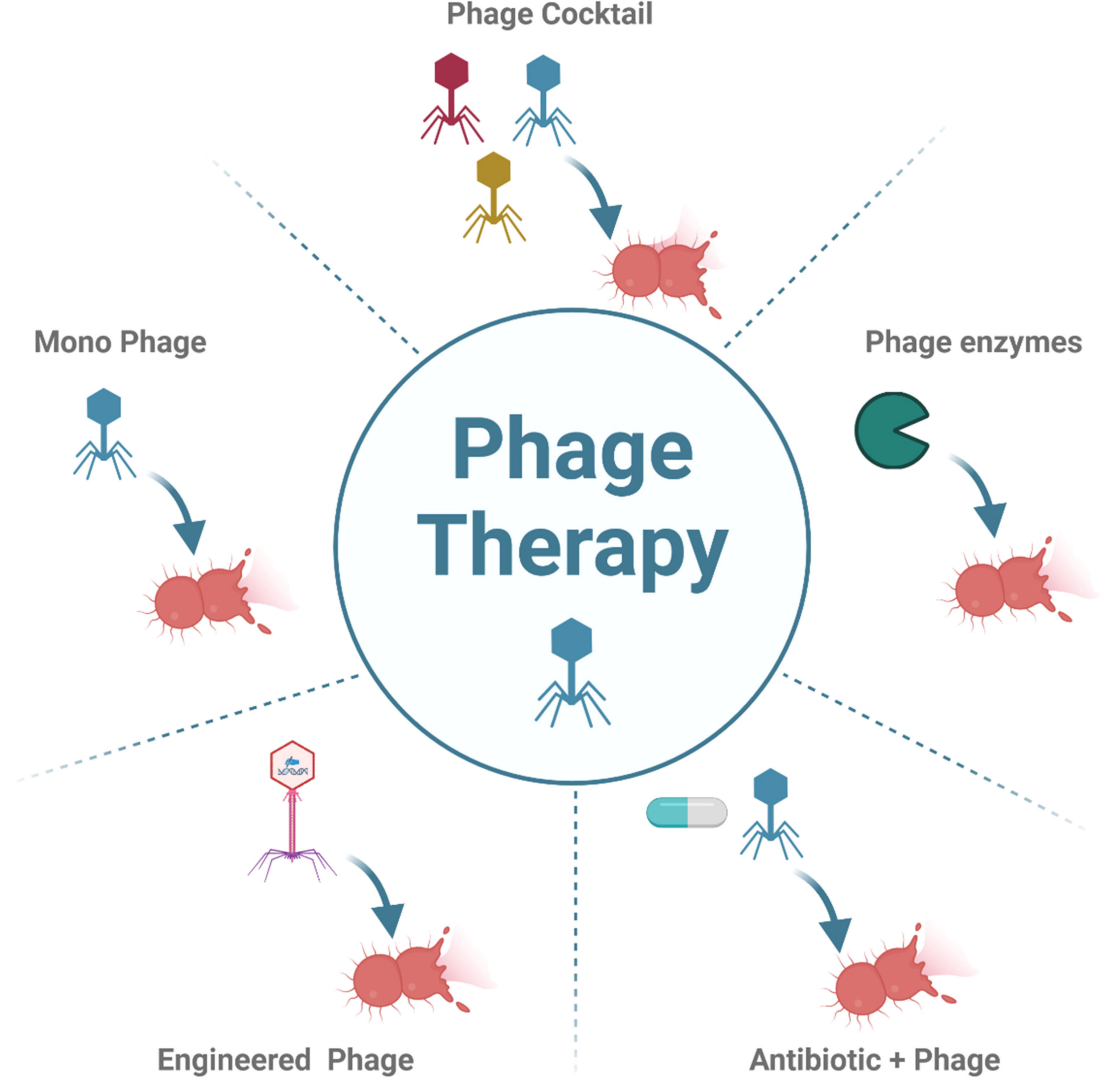
Phage therapy approaches. There are several approach of phage therapy such as Phage cocktail, Mono-phage, engineered phage, combination between antibiotic and phages and phage derived enzymes. Created in BioRender www.biorender.com.

In summary, the implementation of phage therapy using phage cocktails, combination treatments with antibiotics and phages, phage-encoded enzymes, and engineered phages represents the leading alternatives for targeting multidrug-resistant bacteria (**Figure 3**). Even though solid evidence supports the efficacy of these approaches, further trials and investigations are necessary to better understand the perspectives and challenges facing phage therapy.

## 6 Advantages of bacteriophage therapy

Bacteriophages can provide benefits to traditional antibiotics during the current antimicrobial resistance (AMR) emergency. Phages show host preferences as they attack specific bacterial strains which helps preserve microbiota stability and reduces dysbiosis better than broad-spectrum antibiotics that cause microbial equilibrium disruption (Cusumano et al., 2025). Their selectivity also minimizes adverse effects (Cusumano et al., 2025).Phages develop alongside bacteria through co-evolution which helps them defeat numerous bacterial resistance strategies (Hasan and Ahn, 2022). Bacterial resistance to phages can results in the return of antibiotic susceptibility which indicates that resistance development is limited by an evolutionary trade-off (Castledine et al., 2022; Zulk et al., 2022; Anastassopoulou et al., 2024).

Phages combat bacterial biofilms through depolymerase enzymes that break down biofilm matrices which results in enhanced treatment outcomes for recurring infections (Topka-Bielecka et al., 2021; Guo et al., 2023). The self-replicating aspect of phages at infection sites results in extended therapeutic effects and reduces the need for multiple drug applications (Lou et al., 2023; Mishra et al., 2024). Phage-based CRISPR systems also offer benefits through their broad bacterial targeting capabilities and their potential to achieve high efficiency rates. These systems also face limitations as they may only target specific bacteria and production variability remains high necessitating the development of optimized phage libraries, host-range prediction systems and standardized manufacturing procedures.

Phages are considered generally safe and have received GRAS (Generally Recognized as Safe) status (Iacumin et al., 2016). Moreover, a systematic review of safety and toxicity data from 69 publications concluded that serious adverse events were extremely rare, attesting to the safety of phage therapy (Liu et al., 2021). The review showed the effective use of phage cocktails in treating complex infections involving multiple bacterial strains. Bacteriophage toxicity was reported to be low, with most effects being minor and short-lived. Phage therapy appeared to be well tolerated, with no notable immune reactions reported (Liu et al., 2021). Although generally safe, potential immunogenicity and horizontal gene transfer require careful monitoring.

## 7 Evidence of bacteriophage therapy

The emergence of multidrug-resistant (MDR) bacteria has sparked renewed interest in phage therapy. Accordingly, several promising case reports, as well as *in vitro* and *in vivo* studies, have shown the efficacy of phage therapy (**Table 2**).

**Table 2:** Summary of human treatment with bacteriophage therapy.

Phage	Target bacteria	Disease	Treatment and outcome	Reference
3-phage cocktail	*Mycobacterium abscessus*	Cystic fibrosis, chronic *M. abscessus* infection prior to and following bilateral lung transplantation.	A phage cocktail diluted in Phosphate buffered saline to 10^9^ p.f.u./ml was administered intravenously every 12 hours for at least 32 weeks, along with a topical application of 10^9^ p.f.u. in 7 ml to skin lesions. The treatment was well tolerated and restored liver and lung function, as well as healing of skin and surgical sites.	(Dedrick et al., 2019)
32 different bacteriophages marketed as “Otophag”	Multi-bacterial species (Bacteroides spp., Escherichia coli spp., Haemophilus influenzae spp., Klebsiella spp., Moraxella catarrhalis, Morganella morganii, Neisseria spp., Prote- us vulgaris spp., Providencia rettgeri spp., Pseudomonas aeruginosa spp., Staphylococcus aureus spp., Streptococcus pyogenes spp)	Patients with chronic rhinosinusitis with nasal polyps	A gel containing 32 distinct bacteriophages was applied intranasally following endoscopic surgery. This treatment restored the microbial balance in the nasal cavity and reduced inflammation, as evidenced by decreased IL-1β levels and reduced populations of microorganisms, particularly *Enterobacteriaceae*.	(Dobretsov et al., 2021)
Ab_SZ3 phage	Carbapenem-resistant *A. baumannii*	Hospital-acquired pneumonia	The patient received 16 days of continuous nebulized single-phage therapy in combination with tigecycline and polymyxin E, resulting in improved lung function and successful pathogen clearance with well-tolerated treatment.	(Tan et al., 2021)
Customized phage therapy (12 cases)	*Multidrug-resistant bacteria (* *E. coli, S. aureus*, *Klebsiella pneumoniae, Enterobacter cloacae, Klebsiella* *aerogenes, P. aeruginosa, and Enterococcus faecium.*	Diverse infections (bacteremia, osteomyelitis, joint, urinary tract, and respiratory infections)	66% showed favorable responses; 42% achieved bacterial eradication; and no major adverse reactions were reported.	(Green et al., 2023)
Anti-staphylococcal phage	*Staphylococcus aureus*	Diabetic foot infection	Anti-*S. aureus* phage therapy was administered to 10 high-risk amputation patients. The treatment was effective in 9 patients; 1 patient did not respond.	(Young et al., 2023)
Personalized cocktail	*P. aeruginosa* (Pa_AR1), a strong biofilm producer	Chronic right hip prosthesis infection	Phage therapy administered at 10³ p.f.u./ml alongside meropenem resulted in no further adverse reactions after phage volume reduction, with no pain or local inflammation at the hip and no signs of infection relapse; the patient remained in good clinical condition.	(Pirnay et al., 2024)
Staphefekt SA.100, a recombinant phage endolysin	*S. aureus* (both methicillin-sensitive and methicillin-resistant)	Chronic *S. aureus*-related dermatoses	Patients with chronic and recurrent *S. aureus*-related dermatoses were treated topically with Staphefekt SA.100, which, due to its specific mechanism of action, did not induce bacterial resistance.	(Totté et al., 2017)
A single phage	*Achromobacter* *species*	Cyctic fibrosis	IV phage therapy for 28 days (14 days without antibiotics and 14 days with antibiotics) Achromobacter sp. could not be recovered up to 16 weeks after treatment	(Faruk et al., 2025)
HP3, ES17, HP3.1, ES19 phages	*Escherichia coli*	prostate and urinary tract infections	10^9^ PFU/mL every 12h administrated intravenously for 2 wk No adverse effects observed	(Terwilliger et al., 2021)
K, SA4 phages	*Staphylococcus aureus*	recurrent bacteremia, Left Ventricular Assist Device (LVAD)-related infection	10^10^ PFU/mL every 12h administrated intravenously for 6 wk and 3 × 10^10^ PFU/mL administrated intraoperatively once No adverse effects. 19 months after completion of phage therapy, The patient successfully received a heart transplant, and all antibiotics used to treat the infection were discontinued.	(Green et al., 2023)
6917, 6959	*Pseudomonas aeruginosa*	Persistent (dissemination) LVAzD driveline	10^10^ PFU/mL every 12h administrated intravenously for q12h, IV, 6 weeks and 10^11^ PFU/mL administrated intraoperatively once. No adverse effects. The patient still has a small amount of drainage that tests positive for *P. aeruginosa*, but it is managed with local wound care. CT imaging shows no signs of infection. The patient has been off antibiotics since 2022 and remains stable.	(Green et al., 2023)
Intesti and Ses bacteriophages	*E. coli*	chronic bacterial prostatitis	The patient received treatment through different routes (oral, rectal suppository, and intravesical) for 10 days at first. Symptoms improved a lot within 7 days and disappeared completely after a second treatment course. Follow-up cultures showed no bacterial growth, and the patient stayed symptom-free for 4 years.	(Johri et al., 2023)
Phage cocktail	*Klebsiella pneumoniae*	Sternal wound infection (mediastinitis) following aortic arch surgery	The Patient remains infection-free after two years follow-up IV and local administration	(Hameed et al., 2024)
Phage COP-80B	*Staphylococcus epidermidis*	chronic orthopaedic infection	Phage therapy administered with successful outcome. the patient remained infection-free at the 6-month follow-up.	(Munteanu et al., 2025)

For example, a case involving a successful treatment with a phage cocktail on a critically ill one-year-old girl with a vancomycin-resistant *Enterococcus faecium* infection showed clinical improvement with no adverse effects reported (Paul et al., 2021). Similarly, a successful phage cocktail therapy targeting MDR *P. aeruginosa* infection was documented in a 3-month-old girl with severe bronchitis, culminating in her complete recovery (Morozova et al., 2024). Additionally, several studies have proven the impact of phages on certain bacteria, particularly biofilm-forming ones, which tend to resist antibiotics (Suh et al., 2023; Vaezi et al., 2024; Zalewska-Piątek and Nagórka, 2025). Moreover, experimental models show promising results of phages that not only reduced bacterial loads but also increased survival rates in infected animals (Melo et al., 2020). The combination of phages and antibiotics usually enhances treatment efficacy. Furthermore, phage therapy has been shown to alter bacteria’s AMR profiles, rendering them sensitive to antibiotics (Morozova et al., 2018). Nevertheless, more research is needed to optimize phage therapy protocols, including phage pharmacokinetics, dosing regimens, and the production of bioengineered phages and phage endolysins (Palma and Qi, 2024). Moreover, consistent manufacturing standards and regulatory frameworks are critical to ensure batch reproducibility and global clinical acceptance of phage-based therapeutics (Bretaudeau et al., 2020).

## 8 Antibiotic resistance in Saudi Arabia

Reflecting global trends, antibiotic resistance in Saudi Arabia has become a significant public health concern in recent years. This alarming trend of AMR has been observed in Saudi Arabia among various bacterial species. A systematic review of antibiotic resistance trends among *Enterobacteriaceae* in Saudi Arabia was conducted between 2003 and 2023, identifying 7,592 isolates as resistant bacteria (Arafah, 2024). These resistant strains, such as *Escherichia coli* and *K. pneumoniae*, have been associated with a range of clinical conditions, including urinary tract infections, blood stream infections, surgical site infections, and pneumonia. Furthermore, a retrospective study conducted between 2016 and 2019 in a tertiary hospital in Jeddah city showed high levels of AMR among both Gram-negative and Gram-positive bacteria, including *E. coli*, *K. pneumoniae*, *P. aeruginosa*, and *E. faecalis* (Hakami et al., 2022).

*E. coli* is one of the most frequently isolated resistant bacteria, particularly in urinary tract infections and shows high levels of resistance to ampicillin and ceftriaxone (Hakami et al., 2022). For respiratory tract infections, *P. aeruginosa* is among the frequently isolated species, exhibiting high levels of resistance to piperacillin/tazobactam and ciprofloxacin (Hakami et al., 2022). Extended-spectrum beta-lactamases-producing bacteria are major contributors to the AMR crisis, particularly in *E. coli*, and are predominant among resistant pathogens, with some reports indicating a prevalence of up to 87% (Alqasim et al., 2018; Arafah, 2024). Moreover, the most identified genes for ESBL were CTX-M and TEM (Arafah, 2024). Resistance to carbapenems is also a concerning issue in the fight against antibiotic-resistant bacteria, such as *Klebsiella* and *Pseudomonas* (Karakonstantis et al., 2020). OXA-48 is the most frequently identified carbapenemase gene and is responsible for carbapenem-resistant *Enterobacteriaceae* (Arafah, 2024). Additionally, a multicenter study in Saudi Arabia focusing on antibiotic resistance trends in nonfermenting gram-negative bacilli between 2011 and 2016 showed *P. aeruginosa* (73.6%) followed by *A. baumannii* (21.0%) as the most frequently isolated species (Somily et al., 2021). An increased resistance trend was found for *P. aeruginosa* against aztreonam, imipenem, and meropenem, while *A. baumannii* showed complete resistance to several beta-lactam antibiotics and increased resistance to other antibiotics such as tigecycline and tobramycin (Somily et al., 2021).

Regarding pneumonia, a systematic review found that *P. aeruginosa* and *A. baumannii* were frequently isolated from hospital-acquired pneumonia, while *S. aureus* and *Streptococcus* spp. were responsible for community-acquired pneumonia, with increased resistance observed against cephalosporins and carbapenems (Alshammari et al., 2023). Moreover, a 2024 study on *P. aeruginosa* resistance in Saudi Arabia, based on samples collected between 2022 and 2023, revealed that 22.2% of the isolates were classified as difficult-to-treat and 5.4% as pandrug-resistant, indicating that *P. aeruginosa* is a primary healthcare threat pathogen (Thabit et al., 2024). The COVID-19 pandemic has also exacerbated the AMR crisis in Saudi Arabia by increasing the rates of methicillin-resistant *S. aureus* and MDR levels among Gram-positive bacteria during 2020–2021 (Alabdullatif, 2024).

In response to the AMR challenges, Saudi Arabia initiated its second National Action Plan on AMR for 2022–2025, following its first plan in 2017 (MOH, 2022). The second plan adopts a comprehensive strategy that updates and improves the first plan by creating a new AMR national committee to better manage the plan. The second plan employs a One Health approach, encompassing human, animal, and environmental health in combating AMR. With 13 initiatives and 44 activities, the plan focuses on 5 main objectives: strengthening interdisciplinary collaboration, improving AMR awareness, enhancing surveillance and research, optimizing antimicrobial use in both human and animal health sectors, and reducing infection incidence through improved hygiene and prevention measures. Internationally, Saudi Arabia hosted the 4th Global High-Level Ministerial Conference on AMR in 2024, resulting in the Jeddah Commitments (MOH, 2024). One key commitment is to establish an AMR One Health Learning Hub, which focuses on enhancing capacity building and training, particularly in low- and middle-income countries, in collaboration with the WHO Academy. The hub aims to foster a One Health approach (addressing human and animal health, agriculture, and the environment) by supporting the implementation of National Action Plans. Another important commitment from the conference was the creation of a Regional AMR Access and Logistics Hub based in Saudi Arabia, which seeks to improve procurement processes and address challenges related to accessing safe, effective, and affordable antimicrobials and diagnostics. A third commitment is to establish, by 2025 an Independent Panel for Evidence on Action Against AMR, aiming to provide impartial assessments of the AMR impact, prevention strategies, and future risks, guiding global efforts to mitigate the AMR crisis. This conference clearly demonstrates Saudi Arabia’s strong and determined intention to tackle the AMR crisis.

These national efforts show strong commitment to tackle AMR issue but various challenges persist. The surveillance is limited by both the restricted genomic integration and the existing diagnostic inequalities. Better genomic reporting systems and standardized AMR monitoring systems will enhance both policy effectiveness and data accuracy. The ability of Saudi Arabia to monitor resistance patterns and develop specific antimicrobial approaches will improve through increased regional research partnerships and bioinformatics infrastructure development.

## 9 Bacteriophage therapy in Saudi Arabia

Given the antibiotic resistance crisis in Saudi Arabia, the development and implementation of promising alternative treatments such as phage therapy are essential for addressing this public health issue. To enable this agenda, several challenges must be addressed, including further research, education, and economic and accessibility concerns related to phage therapy. Regulatory hurdles are also need to be addressed to exploit the advantages of phage therapy. The Saudi Food and Drug Authority (SFDA) has not yet established specific guidelines for phage therapy approval and manufacturing oversight and clinical implementation which restricts compassionate use protocols for severe or untreatable cases similar to the United States and Belgium regulatory models (Yang et al., 2023). The development of phage therapy in Saudi Arabia and the fight against AMR bacteria would also contribute to the global effort against the AMR crisis. Notably, interest in the field of bacteriophages is increasing in Saudi Arabia, as evidenced by several phage-related publications involving Saudi scientists (Al-Zubidi et al., 2019; Imam et al., 2019; Alsaadi et al., 2022; García-cruz et al., 2023). In 2023, the King Abdullah International Medical Research Center in Riyadh hosted the first Bacteriophage Symposium, providing an opportunity for scientists and clinicians to engage in discussions on the potential applications and future prospects of phage therapy in Saudi Arabia (KAIMRC, 2023). This growing interest in bacteriophage research underscores the necessity of investment in this field, which will play an important role in addressing the antibiotic resistance crisis both nationally and globally.

The research on phages in Saudi Arabia has shown advancement but the field remains scattered because institutions do not work together effectively and the country lacks proper facilities for big-scale production and testing. The development process would speed up through national phage database implementation which connects to centralized biobanks and research networks. The integration of these initiatives with the National AMR Action Plan and One Health framework will enable Saudi Arabia to become a leading country in the Middle East for phage-based therapeutic development and translational research.

## 10 Conclusion

The worldwide threat of antibiotic resistance requires immediate collective action to address this critical issue. The global fight against multidrug-resistant pathogens has not fully succeeded in preventing these pathogens from making several antibiotics ineffective. The use of bacteriophages as a treatment method shows promise as it targets specific bacteria while working better with conventional antibiotics. Saudi Arabia demonstrates its dedication to fight AMR through national plans and international leadership; however, phage therapy development remains at an initial stage. The development of phage therapy faces multiple barriers due to insufficient infrastructure, missing regulations and insufficient clinical guidelines. The future research agenda should include three main objectives: creating national phage banks, linking genomic surveillance to bacterial resistance databases and performing clinical trials of phage-antibiotic treatment combinations. The development of clinical applications for phage therapy requires both enhanced bioinformatics capabilities and expanded institutional partnerships. The One Health-based antimicrobial resistance strategy of Saudi Arabia can be strengthened through the implementation of bacteriophage therapy as a practical and enduring solution.
